# A randomised controlled trial of breast cancer genetics services in South East Scotland: psychological impact

**DOI:** 10.1038/sj.bjc.6601170

**Published:** 2003-08-12

**Authors:** A Fry, A Cull, S Appleton, R Rush, S Holloway, D Gorman, R Cetnarskyj, R Thomas, J Campbell, E Anderson, M Steel, M Porteous, H Campbell

**Affiliations:** 1 Cancer Research UK, Edinburgh Oncology Unit, Western General Hospital, Crewe Road South, Edinburgh EH4 2XR, UK; 2 Department of Clinical Genetics, Molecular Medicine Centre, Western General Hospital, Crewe Road South, Edinburgh EH4 2XU, UK; 3 Lothian NHS Board, Deaconess House, 148 Pleasance, Edinburgh EH8 9RS, UK; 4 Edinburgh Breast Unit, Western General Hospital, Crewe Road South, Edinburgh EH4 2XU, UK; 5 School of Biology, University of St Andrews, St Andrews, Fife KY16 9TS, UK; 6 Department of Public Health Sciences, University of Edinburgh Medical School, Teviot place, Edinburgh EH8 9AG, UK

**Keywords:** breast cancer genetic risk counselling, service delivery, psychological impact

## Abstract

This study compared the psychological impact of two models of breast cancer genetics services in South East Scotland. One hundred and seventy general practices were randomised to refer patients to the existing standard regional service or the novel community-based service. Participants completed postal questionnaires at baseline (*n*=373), 4 weeks (*n*=276) and 6 months (*n*=263) to assess perceived risk of breast cancer, subjective and objective understanding of genetics and screening issues, general psychological distress, cancer worry and health behaviours. For participants in both arms of the trial, there were improvements in subjective and objective understanding up to 4 weeks which were generally sustained up to 6 months. However, improvements in subjective understanding for the women at low risk of breast cancer (i.e. not at significantly increased risk) in the standard service arm did not reach statistical significance. Cancer worry was significantly reduced at 6 months for participants in both arms of the trial. The two models of cancer genetics services tested were generally comparable in terms of the participants' psychological outcomes. Therefore, decisions regarding the implementation of the novel community-based service should be based on the resources required and client satisfaction with the service.

Media attention to scientific developments in cancer genetics has resulted in a greatly increased demand for cancer genetics services. These services aim to identify individuals who have inherited a significantly increased risk of cancer in order to counsel them about their risks and to offer appropriate risk management to reduce morbidity and mortality. There is a challenge to provide this information in ways that the lay public can utilise to inform their health-care choices without causing undue psychological distress. Individuals who are not at significantly increased risk also need appropriate reassurance without precluding an appropriate vigilance to symptoms of sporadic cancer. There is also a challenge to respond to these new developments within existing health-care budgets. Internationally, there is a lack of consensus about how best to deliver cancer genetic services (Steel *et al*, 1999) and an urgent need for empirical evidence to inform service development.

A survey of 22 regional cancer genetics services in the UK in 1998 reported that the predominant users of these services were women with a family history of breast cancer (Wonderling *et al*, 2001). Of the women who are diagnosed with breast cancer, about 10% report having a family history of the disease (Narod, 2002). Of these cases, only a small proportion will be due to inherited genetic mutations in one of the known susceptibility genes, BRCA1 and BRCA2. These genetic mutations give rise to increased lifetime risks of developing the disease, often at an earlier age than is the norm for sporadically occurring cases.

Brain *et al* (2000) showed that there was no difference in the effectiveness of multidisciplinary cancer genetics teams and breast surgeons in terms of psychological outcomes in the management of familial breast cancer in Wales. Secondary analysis of the data (Brain *et al*, 2002) showed some significant differences in psychological outcomes between groups of women at different levels of breast cancer risk. Only those women at low or moderate risk showed significant reductions in cancer worry and perceived risk of breast cancer. Satisfaction with genetic counselling was significantly lower in those women found to be at high risk of breast cancer.

In South East Scotland, a multidisciplinary clinic offering specialist cancer genetic risk counselling and screening to women with a family history of breast cancer has been held in the regional breast screening centre in Edinburgh since 1992. With growing waiting lists for the South East of Scotland familial breast cancer clinic, more stringent referral criteria were applied. GPs referring women judged to be at low risk were sent a letter explaining that no appointment could be offered when the criteria were not met. Referrals of women at relatively low risk were still accepted where the woman's presentation remained a particular cause of concern (e.g. high level of anxiety about breast cancer risk which was difficult for the GP to manage).

An alternative model of cancer genetics services has been proposed (Campbell *et al*, 1995) whereby genetics nurse specialists could offer clinics within GP locality areas to carry out risk assessment, provide counselling for those whose risk was not significantly increased and mediate referral of those at higher risk to the specialist service. It was hoped this would provide improved support to primary care and better services for those at lower risk while encouraging more cost-effective use of specialist resources for those at increased risk of developing breast cancer.

We carried out a cluster randomised trial of this new model of service delivery comparing it to the existing multidisciplinary specialist service. This paper presents a comparison of the psychological outcomes of these two service models and across participant's level of breast cancer risk.

## MATERIALS AND METHODS

### Participants

Ethical approval for the study was obtained from the local ethics committee. An invitation to take part in the trial was sent to all general practices in Lothian (*n*=125), South West Fife (*n*=54) and Borders (*n*=24) Health Boards in South East Scotland. One hundred and seventy practices (84%) agreed to take part, 23 (11%) declined and 10 (5%) did not reply. This meant that 725 of the 828 (88%) GPs in practice across these three Health Boards agreed to refer patients into the trial. Practices were randomly assigned to either arm of the trial using a minimisation technique (Pocock, 1983, pp 84–86) to ensure that the two groups were balanced for size of practice, historical referral rate and social deprivation index.

During the period March 1998 to November 1999, any woman referred from participating GP practices to the regional clinical genetics department for breast cancer genetic risk counselling was invited to take part in the trial. To be eligible for the trial, women had to live in the region, be able to give informed consent and had to complete a baseline questionnaire. Women who were symptomatic or had been diagnosed with breast and/or ovarian cancer were excluded from the trial as were those who had previously consulted another clinic about their family history of cancer. Those who were ineligible to participate were offered the standard regional service.

### Procedure

Potential participants were sent an information sheet and were invited to return the consent form to indicate whether they were willing to participate in the trial. Those who consented were then asked to complete a baseline questionnaire. Reminders were sent to all nonresponders after approximately 4 weeks. Only those who completed the baseline questionnaire were enrolled in the trial. Nonresponders and those who did not consent to participate in the trial were offered the standard regional service. The service offered to women who returned a completed baseline questionnaire was dependent on the arm of the trial to which their GP practice had been randomised.

#### Standard (regional) service

Women were sent a family history form and a baseline questionnaire to complete. The family history form requested information about first-, second- and third-degree relatives. If the family history form was not returned, a letter was sent to the woman and to her GP to explain that no consultation was possible without this information. A genetics consultant (MS) and genetics nurse specialist (JC) assigned categorical risk assessments informed by published criteria (Table 1

**Table 1:** Criteria for assessing a significantly increased risk of breast cancer (Cancer Research Campaign, 1997)

A woman's risk of developing breast cancer is moderately increased if she has one of the following:
• A first-degree relative with breast cancer diagnosed under 40 years.
• Two first- or second-degree relatives on the same side of the family with breast cancer diagnosed under 60 years or with ovarian cancer.
• Three first- or second-degree relatives on the same side of the family with breast or ovarian cancer.
• A first-degree relative with breast cancer in both breasts.
• A first-degree *male* relative with breast cancer.

) using the information on the completed family history form. If necessary, further information and/or confirmation of relatives’ diagnoses were obtained by a genealogist and from the Scottish Cancer Registry. When a woman was assessed as being at ‘low risk’ (i.e. not at significantly increased risk), she and her GP were sent a letter to explain this. Women assessed as being at ‘moderate’ or ‘high risk’, or where an adequate risk assessment could not be made from the information available, an appointment at the familial breast cancer clinic were offered. The clinic consultation offered more detailed discussion with a genetics consultant about risk status and with a specialist breast surgeon about options for risk management (i.e. breast cancer screening and, for ‘high-risk’ women, prophylactic mastectomy or chemoprevention). Clinical breast examination and mammography (where appropriate) were carried out at this visit. After this appointment, the patient's GP was sent a letter to summarise the issues discussed. All women were asked to complete a postal follow-up questionnaire 4 weeks and 6 months later.

#### Novel (community-based) service

All women in this arm of the trial were sent an initial appointment for one of the community-based clinics (held in a GP practice near to where they lived), run by a genetics nurse specialist (RC/RT). At the clinic, the genetics nurse specialist ascertained the woman's family history of cancer and compiled a family tree. This information was compared to published criteria (Table 1) to determine whether she was at significantly increased risk. When an adequate risk assessment could not be made during the appointment, further information and/or confirmation of relatives’ diagnoses were obtained from the patient or medical records, before the patient was informed of their risk by letter. Women deemed not to be at significantly increased risk (i.e. in the ‘low-risk’ category) were offered information and reassurance and were discharged from the clinic. These patients and their GPs were sent a letter reaffirming their ‘low-risk’ status and summarising the issues discussed at the appointment. Women found to be at increased risk (i.e. in the ‘moderate-risk’ or ‘high-risk’ categories) were offered an appointment at the regional centre with a geneticist and genetics nurse specialist. All women were asked to complete a postal follow-up questionnaire 4 weeks and 6 months later.

### Sociodemographic and objective breast cancer risk data

Women were asked to record their date of birth, marital status and educational level on the baseline questionnaire. Information about the category of breast cancer risk to which each woman had been assigned was derived from the clinical records.

### Psychological measures

#### Subjective understanding

Women were asked to rate on a 4-point scale from 1 (*not at all*) to 4 (*very much*) how well they understood each of four issues relevant to breast cancer genetic risk. The issues were:

1. How increased risk of breast cancer is passed on in families.
2. The significance of their own family history of cancer.
3. Whether there was anything they could do to reduce their risk of developing breast cancer.
4. What services were available to protect the health of people at increased risk of breast cancer.

Responses were summed to give a composite score for subjective understanding ranging from 4 to 16.

#### Objective understanding

Participants were asked to consider a number of factual statements and to respond ‘true’, ‘false’ or ‘don't know’. There were 10 statements about breast cancer genetics (e.g. ‘Only a parent who has had breast cancer can pass on increased risk to their children’) and 12 statements about issues surrounding mammography (e.g. ‘Mammograms are used to detect early stages of breast cancer’). Statements were scored 1 (*correct*) or 0 (*incorrect/don't know*), and the number of correct responses combined to give total scores for *genetics understanding* (range 0–10) and *mammography understanding* (range 0–12).

#### Perceived risk of breast cancer

Although a number of items were used to assess perceived risk of breast cancer, the results of one item were analysed for the purposes of this report. Participants were asked to indicate whether they considered their own level of risk to be *high*, *moderate* or *low*.

#### Psychological distress

*General Health Questionnaire 30-item version (GHQ-30)* (Goldberg and Williams, 1988). This well-validated scale was scored using the GHQ method (0, 0, 1, 1) using a threshold of ⩾6 to screen for ‘case-level’ general psychological distress.
*Cancer Worry Scale* (Watson *et al*, 1998). This six-item scale (adapted from four single items, Lerman *et al*, 1991a,1991b) assesses concerns about developing cancer and their impact on daily functioning. Total scores range from 6 to 24 where a higher score indicates higher levels of worry. The psychometric properties of the scale have been shown to be satisfactory (Brain *et al*, 1999; Hopwood *et al*, 2001).

#### Health behaviours

Several *ad-hoc* items indicated the extent to which genetic counselling may have influenced the women's health behaviour. Participants at 4 weeks were asked retrospectively about their health behaviours prior to counselling (i.e. the frequency of breast self-examination, smoking, drinking alcohol, trying to lose weight, eating bran and high-fibre foods, avoiding fatty foods, eating a balanced diet, taking exercise, looking after their health in general). They were asked to rate whether the frequency of any of these behaviours had changed since consulting genetics services (at 4 weeks) or in the last 6 months (at 6 months) on a scale from 1 (*much less than before*) to 5 (*much more than before*).

### Statistical methods

Descriptive statistics were generated to describe the study participants. Differences between two independent groups were analysed with independent samples *t*-tests (two-tailed), Mann–Whitney, *χ*^2^ (two-tailed) or Fisher's exact tests (two-tailed). A 2 (trial arm) × 2 (objective risk) repeated measures analysis of variance (ANOVA) was used to determine between-group differences and within-group changes over time (baseline, 4 weeks, 6 months) in psychological outcomes and possible interactions between trial arm, objective risk and time. Significant effects were followed up with *post-hoc* tests (independent samples *t*-tests, paired *t*-tests, ANOVA). *χ*^2^ (two-tailed), Fisher's exact (two-tailed), Cochran's and McNemar tests were used to examine the impact of time, trial arm and objective risk on perceived risk and the proportion of participants suffering from ‘case-level’ distress. A significance level of 0.05 was used throughout. The data were analysed using SPSS for Windows version 10.00 (1999).

## RESULTS

### Participants

Figure 1

**Figure 1:**
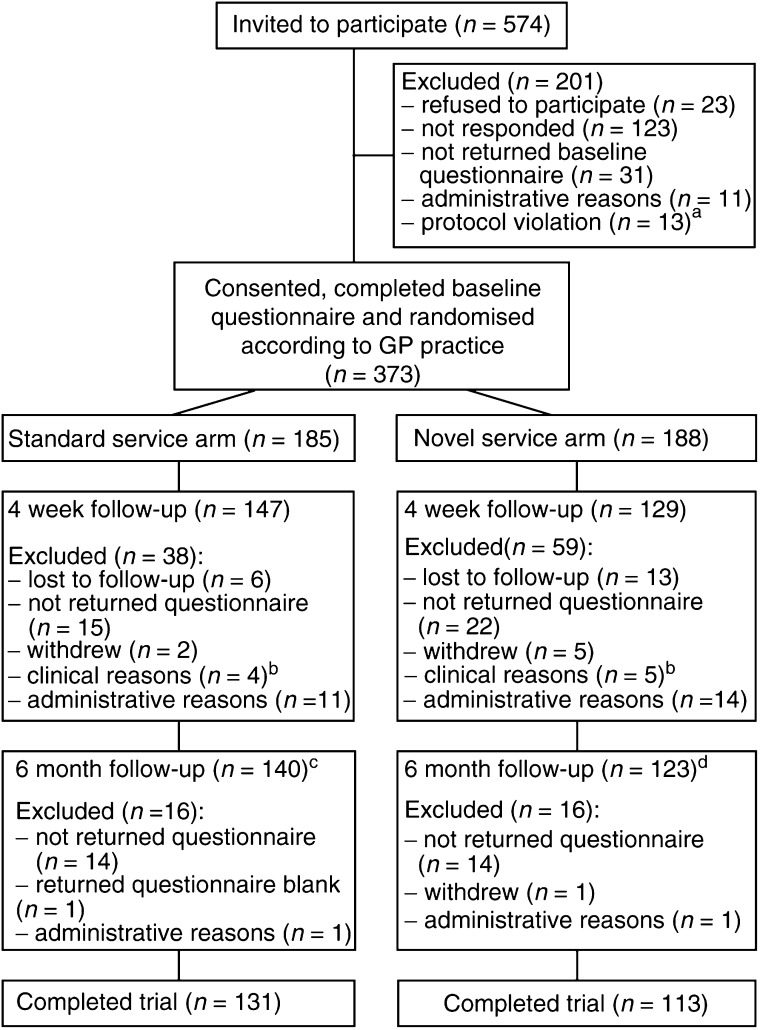
Progress of participants through the trial. ^a^ For example, the women had received genetic counselling elsewhere or had been treated for cancer. ^b^ For example investigation of breast symptoms. ^c^ Includes nine women who were excluded at the 4 week assessment due to administrative reasons (*n*=5) or nonreturn of the questionnaire (*n*=4). ^d^ Includes 10 women who were excluded at the 4 week assessment due to administrative reasons (*n*=4) or nonreturn of the questionnaire (*n*=6).

shows the progress of participants through each arm of the trial.

#### Baseline

Over the study period, 574 women, referred for breast cancer genetic risk counselling, were invited to participate in the trial. Consent forms were returned by 451 women (response rate 79%), of whom 428 (75% of those invited) agreed to participate in the study. Three hundred and seventy-three of these women (87% of those who consented) returned a completed baseline questionnaire, 185 of whom were then assigned to the standard service arm and 188 to the novel service arm of the trial according to their GP practice.

#### 4 weeks

Of the 373 women who completed a baseline questionnaire, 276 also completed a 4 week follow-up questionnaire (74% of those who were enrolled in the trial), 147 from the standard service arm and 129 from the novel service arm of the trial.

The characteristics of those for whom only baseline data were available (‘baseline only group’; *n*=97) were compared with those of the ‘4 week group’ (*n*=276) to check for participation bias. A significantly greater number of the ‘baseline only group’ had been assigned to the novel service arm than the standard service arm of the trial (61 *vs* 39%; *χ*^2^=5.70, df=1, *P*=0.018). A higher proportion of women in the ‘baseline only group’ were categorised as being at low risk (54 *vs* 32%; *χ*^2^=14.01, df=1, *P*<0.000). Similarly, a greater proportion of women in this group were suffering from ‘case-level’ distress at baseline (43 *vs* 31%; *χ*^2^=4.53, df=1, *P*=0.043). The ‘baseline only group’ had significantly higher scores at baseline on the Cancer Worry Scale (mean=12.18/11.10; s.d.=3.29/2.98; *t*=2.97, df=367, *P*=0.003). There were no significant differences between the two groups on any of the other sociodemographic or psychological variables at baseline.

#### 6 months

Two hundred and sixty-three women completed 6 month follow-up questionnaires (71% of those who were enrolled in the trial), 140 women from the standard service arm and 123 from the novel service arm of the trial. This includes 19 women who were excluded at the 4 week assessment due to administrative reasons (*n*=9) or nonreturn of the questionnaire (*n*=10). The baseline characteristics of those for whom the 6 month questionnaires were analysed (‘6 month group’; *n*=263) were compared with those participants who completed the 4 week questionnaire but not the 6 month questionnaire (‘Not 6 month group’; *n*=32) to check for participation bias. There were no significant differences between the number of women who dropped out from either arm of the trial. A greater proportion of women in the ‘Not 6 month’ group were classified as being at low risk of breast cancer (53 *vs* 31%; *χ*^2^=6.41, df=1, *P*=0.016). This group scored significantly lower on objective understanding of mammography (*t*=−2.37, df=270, *P*=0.018) and had a tendency to have higher scores on the Cancer Worry Scale (*t*=1.83, df=290, *P*=0.068). There were no significant differences between the two groups on any of the other sociodemographic or psychological variables (marital status and perceived risk could not be analysed due to small numbers in some categories).

### Comparison of trial arms on sociodemographic and objective breast cancer risk characteristics

The sociodemographic characteristics of participants and the breast cancer risk category to which they were assigned are shown in Table 2

**Table 2:** Sociodemographic, objective breast cancer risk and psychological characteristics of the two trial groups at baseline, 4 weeks and 6 months^a^

	**Baseline (*n*=373)**		**4 weeks (*n*=276)**		**6 months (*n*=263)**	
**Variable**	**Standard service (*n*=185)**	**Novel service (*n*=188)**	**Standard service (*n*=147)**	**Novel service (*n*=129)**	**Standard service (*n*=140)**	**Novel service (*n*=123)**
Age (years): mean (s.d.)	37.3 (9.4)	39.1 (9.6)	36.8 (9.4)	39.7 (9.4)*	37.4 (9.5)	39.5 (9)

Marital status: *n* (%)						
Married/cohabiting	130 (71)	136 (73)	104 (72)	100 (79)	98 (71)	93 (77)
Separated/divorced/widowed	24 (13)	24 (13)	18 (13)	14 (11)	20 (14)	16 (13)
Never married	28 (15)	26 (14)	22 (15)	13 (10)	21 (15)	12 (10)

Education: *n* (%)						
To age 16 years	65 (36)	68 (37)	49 (34)	44 (34)	46 (33)	42 (34)
To age 18 years	30 (17)	28 (15)	23 (16)	19 (15)	23 (17)	15 (12)
After age 18 years	46 (26)	50 (27)	35 (24)	37 (29)	35 (25)	37 (30)
University graduate	39 (22)	40 (22)	36 (25)	28 (22)	34 (25)	28 (23)

Risk of breast cancer: *n* (%)						
Objective:						
Low	50 (28)^b^	81 (46)*	34 (23)	53 (41)*	31 (22)	50 (41)*
Moderate/high	129 (72)	97 (55)	113 (77)	76 (59)	109 (78)	73 (59)

Perceived:						
Low	5 (3)	7 (4)	12 (8)	10 (8)	11 (8)	11 (9)
Moderate/high	179 (97)	180 (96)	134 (92)	117 (92)	127 (92)	108 (91)

Understanding: mean (s.d.)						
Subjective^c^	9.4 (2.8)	8.9 (2.5)	12 (2.2)	11.7 (2.3)	11.9 (2.2)	11.7 (2.3)
Objective:						
genetics^d^	3.9 (2.5)	3.9 (2.2)	6.3 (2.3)	6 (2.2)	6.3 (2.2)	6 (2.1)
mammography^e^	7.3 (1.9)	7.0 (1.8)	8 (1.8)	7.8 (1.7)	7.9 (1.8)	7.9 (1.7)

GHQ-30:						
Total score: median (IQR)	2 (9)	2 (7.3)	1 (8)	2 (8.5)	0 (4)	0 (5)
‘Case-level’ distress: *n* (%)^f^	66 (36)	58 (31)	32 (21)	27 (22)	29 (21)	28 (23)

Cancer worry: mean (s.d.)	11.5 (3.2)	11.3 (3.0)	10.3 (2.4)	10.2 (2.7)	9.9 (2.5)	9.7 (2.7)

a Sample size varies due to missing data.

b The majority of women who were assigned a low breast cancer risk in the standard service arm were informed by letter only.

c Possible range of scores: 4–16.

d Possible range of scores: 0–10.

e Possible range of scores: 0–12.

f Scores of ⩾6.

* *P*<0.05.

. A greater proportion of women at low risk of breast cancer were in the novel service arm than the standard service arm of the trial at baseline (*χ*^2^=11.86, df=1, *P*=0.001), 4 weeks (*χ*^2^=10.26, df=1, *P*=0.002) and 6 months (*χ*^2^=10.52, df=1, *P*=0.001). Women with 4 week follow-up data in the novel service arm were somewhat older than those in the standard service arm (*t*=−2.51, df=274, *P*=0.013). There were no significant differences between the trial arms on any of the other sociodemographic variables at the three assessment points.

### Comparison of trial arms on psychological characteristics and changes over time (by trial arm and objective breast cancer risk)

Table 2 shows the psychological characteristics of the women in the two trial arms at baseline, 4 weeks and 6 months. There were no significant differences between the two trial arms on any of these variables at the three assessment points. Table 3

**Table 3:** Repeated measures ANOVA for trial arm × time × objective breast cancer risk on psychological characteristics

**Variable**	**Effect**	***F***	**df**	***P***
Subjective understanding	Trial arm	0.244	1	0.622
	Time	107.82	1.675	0.000**
	Trial arm × time	2.092	1.675	0.133
	Risk	3.915	1	0.049*
	Trial arm × risk	0.032	1	0.859
	Time × risk	6.705	1.675	0.003*
	Trial arm × time × risk	3.234	1.675	0.049*

Objective understanding: Genetics	Trial arm	0.459	1	0.499
	Time	105.741	1.789	0.000**
	Trial arm × time	0.474	1.789	0.601
	Risk	3.31	1	0.07
	Trial arm × risk	0.708	1	0.401
	Time × risk	0.923	1.789	0.389
	Trial arm × time × risk	2.573	1.789	0.084

Objective understanding: Mammography	Trial arm	0.909	1	0.341
	Time	17.713	1.9	0.000**
	Trial arm × time	2.051	1.9	0.133
	Risk	0.857	1	0.356
	Trial arm × risk	0.068	1	0.794
	Time × risk	0.894	1.9	0.405
	Trial arm × time × risk	1.681	1.9	0.189

Cancer worry	Trial arm	0.117	1	0.733
	Time	36.9	1.62	0.000**
	Trial arm × time	0.535	1.62	0.549
	Risk	0.525	1	0.47
	Trial arm × risk	0.001	1	0.97
	Time × risk	0.127	1.62	0.838
	Trial arm × time × risk	2.675	1.62	0.082

* *P*<0.05.

** *P*<0.001.

presents the results of repeated measures ANOVA for the 244 participants (65% of those who were enrolled in the trial) who completed the baseline, 4 week and 6 month questionnaires.

#### Subjective understanding

Overall, there was a significant improvement in subjective understanding during the course of the study. Further analysis showed that subjective understanding only improved to a significant degree between baseline and 4 weeks (*t*=−14.97, df=231, *P*<0.000). Scores on subjective understanding were shown to be dependent on objective breast cancer risk. *Post-hoc* analysis revealed that women at moderate/high risk had significantly greater scores on subjective understanding than those at low risk at 4 weeks (*t*=−2.69, df=235, *P*=0.008) and 6 months (*t*=−2.46, df=109.214, *P*=0.015). The main effects of time and objective risk were modified by a significant interaction between these two factors. Subjective understanding had significantly improved for both women at moderate/high risk (*t*=−13.70, df=164, *P*<0.000) and women at low risk (*t*=−6.55, df=66, *P*<0.000) between baseline and 4 weeks. However, the improvement was significantly greater in women at moderate/high risk than those at low risk (*t*=−2.51, df=230, *P*=0.013). In addition, there was a significant interaction between trial arm, time and objective risk. *Post-hoc* analysis indicated that differences in subjective understanding between the risk groups within the different trial arms were significant only between baseline and 4 weeks (*F*(1, 226)=5.27, *P*=0.023). Between these two time points, the moderate/high-risk women in the standard service arm (*t*=−11.64, df=98, *P*<0.000) and low risk (*t*=−7.32, df=41, *P*<0.000) and moderate/high-risk women (*t*=−7.58, df=65, *P*<0.000) in the novel service arm had made significant improvements in subjective understanding. There were no significant differences in the extent to which subjective understanding had improved between these groups. Although for women at low risk in the standard service arm there was an improvement in subjective understanding between baseline and 4 weeks, this did not reach statistical significance.

#### Objective understanding

There was a significant improvement in objective understanding of genetics and mammography across all participants during the study period. *Post-hoc* tests showed that scores on these two measures had significantly improved between baseline and 4 weeks only (genetics: *t*=−14.37, df=232, *P*<0.000; mammography: *t*=−5.56, df=214, *P*<0.000).

#### Cancer worry

For all participants, there was a significant reduction in scores on the Cancer Worry Scale during the course of the study. *Post-hoc* analysis revealed that the greatest reduction in scores occurred between baseline and 4 weeks (*t*=5.86, df=239, *P*<0.000) with a smaller, but nevertheless significant reduction between 4 weeks and 6 months (*t*=3.05, df=238, *P*=0.003).

#### General psychological distress

Although there was a significant decrease in the overall proportion of participants suffering from ‘case-level’ distress over the study period (Cochran's *Q*=11.44, df=2, *P*=0.003), further investigations showed that the reduction was only significant between baseline and 4 weeks (McNemar *χ*^2^=8.27, *P*=0.004). There were no significant differences in the proportion of women suffering from ‘case-level’ distress between trial arms or risk groups at the three assessment points.

#### Perceived risk of breast cancer

There were significant changes in perceived risk of breast cancer across all subjects over the study period (Cochran's *Q*=10.5, df=2, *P*=0.005). Further analysis showed that these changes were only significant between baseline and 4 weeks where significantly less women perceived their risk as low at 4 weeks (*P*=0.011). There were no significant differences in perceived risk by trial arm at the three assessment points. However, a significantly greater proportion of women at low objective risk of breast cancer than those at moderate/high objective risk perceived their risk to be low at 4 weeks (*χ*^2^=19.94, df=1, *P*<0.000) and 6 months (*χ*^2^=12.24, df=1, *P*=0.002).

### Comparison of trial arms on health behaviours

At 4 weeks, proportionately more women in the standard service arm reported examining their breasts every month as recommended (32 *vs* 23%) and proportionately more women in the novel service arm reported examining their breasts more frequently than once per month (11 *vs* 4%; *χ*^2^=9.86, df=4, *P*=0.043). There were no significant differences between the two trial arms in the extent to which participants reported performing health behaviours prior to genetic counselling or reported a change in these behaviours after counselling. At 6 months, there were no significant differences between the two groups in the proportion of women who reported changing any of their health behaviours in the last 6 months.

## DISCUSSION

The present study responded to an urgent need for empirical evidence to inform the development of cancer genetics services in South East Scotland. A novel community-based service to provide genetic risk counselling for women with a family history of breast cancer was compared to the existing standard regional service.

The initial response rate to invitations to participate was good with 75% of the women invited agreeing to take part in the trial. The participation rates at each assessment point were satisfactory (baseline: 87%; 4 weeks: 74%; 6 months: 71%) with 65% of those enrolled in the trial completing all three questionnaires. The amount of data lost due to administrative reasons was comparable across the trial arms.

Women who dropped out of the study tended to be in the novel service arm of the trial or at low risk of breast cancer. The latter finding is not unexpected since these women may have been less motivated to continuing participating in a study of cancer genetics services which they were ineligible to receive. However, the women who dropped out of the study had greater levels of psychological distress. As these women may have dropped out in an effort to reduce their high levels of distress, they could perhaps benefit from further psychological intervention. Similar findings have been demonstrated by a previous trial of cancer genetics services (i.e. Brain *et al*, 2000). Given these potential participation biases, the results should be interpreted with caution in regard to their generalisability to a wider population.

The cluster randomisation strategy resulted in comparable trial arms at baseline in terms of sociodemographic and psychological characteristics. However, a greater proportion of women in the novel service arm were assigned a low risk of breast cancer. Further investigation is warranted to determine if this finding is due to chance or differences between the trial arms in terms of the method of risk assignment or the accuracy of family history details reported by participants.

At baseline, subjectively rated understanding of issues related to breast cancer genetic risk was relatively low (mean scores=9.4 for the standard service arm /8.9 for the novel service arm out of a possible 16) and this was reflected in the objective assessment of understanding. On average, correct responses were given to about one-third of the breast cancer genetics items and to about two-thirds of the mammography items. About one-third of participants were suffering from ‘case-level’ distress. This is comparable to the findings in other samples of women prior to genetic risk counselling using the same measure and threshold (Cull *et al*, 1999,^2001^) and to published data from the general population (Goldberg and Williams, 1988). Mean scores on the Cancer Worry Scale were similar to those reported in women prior to genetic risk counselling by Watson *et al* (1998) and Brain *et al* (2000) and slightly lower than those reported by Hopwood *et al* (2001) and Bish *et al* (2002).

The findings show that after consulting cancer genetics services, many of the short-term improvements in psychological outcomes experienced across participants were maintained up to 6 months. All participants reported greater subjective understanding of issues related to breast cancer risk and these improvements were most marked up to 4 weeks and were generally sustained up to 6 months. However, for the women at low risk of breast cancer in the standard service arm of the trial, unlike all other participant groups, these improvements did not reach statistical significance. This may be due to the fact that the majority of these women received a letter informing them about their low risk and were not offered a face-to-face consultation. Improvements in subjective understanding across participants were reflected by improvements in objective understanding which were again most evident between baseline and 4 weeks and were commonly maintained at a similar level up to 6 months. Although participants at low risk of breast cancer in the standard service arm did not feel that their subjective understanding had improved as much as the other participants, there were no differences between trial arms or risk groups in significant improvements on objectively assessed understanding.

It was reassuring to find that despite improvements in objective knowledge, the proportion of women suffering from ‘case-level’ distress decreased up to 4 weeks and cancer worry continued to decrease up to 6 months. Unlike previous research that found significant reductions in cancer worry only for those women at low or moderate risk of breast cancer (i.e. Brain *et al*, 2002), reductions in cancer worry in the present study were not dependent on objective risk.

Women's perceptions of their risk of breast cancer were altered during the course of the study with significantly fewer women overall perceiving their risk as low at 4 weeks, than at baseline. More women who were informed about a low risk of breast cancer perceived their risk of breast cancer as low following their risk assessment, as may be expected. Similar findings have been reported elsewhere (Bish *et al*, 2002). This suggests that the accuracy of perceived risk for the low-risk group improved during the course of the study. However, given the fact that responses to only one of the *ad-hoc* items were analysed for the purposes of this report and the accuracy of participants' risk perceptions was not assessed in this study, it is difficult to make any firm conclusions from these results. However, there is no evidence to suggest that learning that your risk of developing breast cancer is greater than you believed prior to genetic risk counselling causes psychological distress (Cull *et al*, 1999; Watson *et al*, 1999).

This study has shown that the novel community-based model of breast cancer genetics services is generally comparable to the existing standard regional service in terms of the psychological outcomes experienced by recipients. Therefore, decisions regarding the implementation of the novel model of services should be based on additional factors such as the resources required and client satisfaction with the service. These factors have been investigated and will be published separately.
